# Post and Core Treatment to Refit Telescopic Crown-Retained Dentures after Abutment Tooth Fracture: An Evaluation of Therapy by Retrospective Survival Analysis

**DOI:** 10.3390/dj12070224

**Published:** 2024-07-19

**Authors:** Jonas Adrian Helmut Vogler, William Abrahamian, Sarah Marie Reich, Bernd Wöstmann, Peter Rehmann

**Affiliations:** Dental Clinic—Department of Prosthodontics, Justus Liebig University, Schlangenzahl 14, 35392 Giessen, Germany

**Keywords:** post and core, telescopic denture, survival time, Cox regression, Kaplan–Meier analysis, retrospective study

## Abstract

Telescopic crown-retained dentures (TCDs) are one of the most common types of prosthetic restorations for partially edentulous patients; however, post and core (PC) treatment shows the worst survival probability if the tooth is used as an abutment for the TCD. Due to extra axial forces, abutment tooth fracture is a common cause of failure for TCDs; thus, PC treatment is often needed to refit the existing telescopic crown (TC). However, there are no clinical survival data on whether the PC treatment was used to refit the TC after abutment tooth fracture (PC2) or the PC was already fitted at the time of TCD treatment (PC1). A total of 246 patients with 399 PC treatments were retrospectively evaluated for follow-ups up to 17.33 years. The files were analysed for PC1 and PC2. Furthermore, the influence of the jaw, type of tooth, luting material, PC material, bone attachment, therapist and cause of failure was recorded. For statistical analysis, Kaplan–Meier and Cox regression analyses were conducted. PC2 showed highly significant lower survival probabilities than PC1 (*p* < 0.001). Moreover, the bone attachment and the age of the patient at the time of fitting the PC crown had an influence on the survival (*p* < 0.001). Therefore, PC2 should be carefully discussed with the patient and PC1 should be favoured in endodontically treated abutment teeth for TCDs.

## 1. Introduction

Telescopic crown-retained dentures (TCDs) are a frequently used treatment option for patients with few remaining teeth [1,2]; however, the survival probability of post and core (PC) treatment under TCDs is the worst compared to all other types of covering prosthetic restorations [3,4,5]. In this context, fracture of abutment teeth is frequently described as one of the most common causes of failure with TCDs [6,7,8]. Due to extra axial forces when the TCD is removed and inserted incorrectly [3], or when the long denture saddle does not fit the edentulous jaw areas [8], the risk for abutment tooth fracture increases because of its rigid connection to the denture [6,8]. Thus, patients treated with TCDs need to follow a strict aftercare in order to avoid early failures that lead to a bad survival probability [7,8]. Fortunately, abutment tooth fracture does not mandatorily lead to extraction of the tooth [9]. Since for TCDs comparably a lot of coronal hard tissue has to be removed [10], fracture often occurs within the area of the preparation for the telescopic crown (TC), resulting in an insufficient coronal height to reattach the existing TC without additional effort [11]. In these cases, tooth preservation is possible, but in order to refit the existing TC, PC fitting after endodontic treatment is needed in order to restore enough retention surface [12]. The precondition for this is that the fracture line is at least 2 mm above the preparation margin for the TC, because this ferrule is known to be the predominant factor for long-term success of both the tooth and the PC treatment [11]. Moreover, tooth preservation after fracture is of high clinical importance since the number of abutments significantly influences the survival because the chewing forces can be better distributed when more teeth remain as abutments for TCDs [6,8].

Furthermore, TCD fabrication is associated with high costs for the patient because of the technically complex workflow [6,8,13]. Therefore, refitting of an existing TC after abutment tooth fracture is economically advantageous because the friction of the TC in the secondary crown of the TCD is hardly adjustable, and thus expensive and time consuming if fabrication of a new TC is needed [13]. Moreover, for the renewal of the TC the patient has to temporarily forgo the denture for adjustment, which is associated with a decrease in both aesthetics and chewing comfort. Nevertheless, abutment tooth fracture can also be associated with microcracks in the root dentine, which in some cases can be undetectable by visual examination [14]. Against the background of extra axial forces on the abutment teeth of TCDs, microcracks as well as the accuracy of fit of PC treatment can have an increased influence on survival because a large cement gap is associated with an increase in microleakage [15] and polymerisation shrinkage of the adhesive cement [16]. This can cause an uneven transmission of force into the root, leading to an enlargement of microcracks and catastrophic root fractures, making extraction of the abutment tooth inevitable [17,18]. Moreover, a bad accuracy of fit of the PC treatment is associated with an increasing risk of decementation of the PC treatment, which can lead to secondary caries and an increasing risk of tooth loss at a later stage [4,19].

Besides that, the influence of co-parameters (e.g., the type of tooth or the PC material) on survival are inconsistently described by different authors in the literature [11,20,21]. Garcia et al. reported that there was no significant difference in the survival rate between anterior and posterior teeth [22], whereas Dittmann et al. found a better survival probabilities for anterior teeth [23]. However, both authors agreed on the necessity for more evidence by more studies with longer follow-ups and larger sample sizes [22,23]. Furthermore, many studies considered the type of covering prosthetic restoration on the survival of the PC treatment and found significantly lower survival rates when the PC crown was fitted under TCDs than under fixed dental prostheses [3,4,5]. However, the authors did not consider if the PC crown was fitted before treatment for TCDs or after fracture of an abutment tooth in order to refit an existing TC. Therefore, one cannot evaluate the treatment of reattaching a TC with PC treatment by the results of these studies. To the best knowledge of the authors, there is no analysis on this in the scientific dental literature considering possibly influencing co-parameters on the basis of a large sample size with long follow-ups. Since abutment tooth fracture is one of the most common complications with TCDs [6,7,8], an evaluation of this treatment option is of high clinical relevance. Therefore, the aim of this study was to investigate the survival probability of PC crowns fitted before treatment for TCDs (PC1) and to compare it to PC treatment refitting an existing TC after abutment tooth fracture (PC2).

## 2. Materials and Methods

This study was approved by the ethics committee of the Justus-Liebig University Giessen (Reg No 164/11). The observation period for the present study was from 2004 to 2023. All patient files within this period were digitally documented, and thus an automatised search for PC treatment was possible. Since 2004, every patient at our clinic is documented using the same software (MZD, Version 3.11.0.1, Department of Prosthodontics JLU Giessen, Germany), with standardised and unchanged evaluation sheets. Therefore, the documentation is consistent between the different users within the whole observation period.

Initially, all patients with PC treatment between 2004 and 2023 (n = 1661) were filtered. The files were subsequently searched for covering prosthetic restorations different to TCDs, lack of data, unstandardised PC workflows and serious systemic illnesses possibly influencing the survival of PC treatment. These files were excluded from further investigation and from the data acquisition of the present study. Furthermore, all patients with TCDs including less than two abutment teeth were excluded from further investigation in this study.

Finally, 246 patient files with overall 399 PC treatments fulfilled the inclusion criteria and were analysed according to a standardised evaluation sheet including the following information and general demographic data such as age and gender of the patient:

- Date of cementation/date of the final observation or failure;
- Reason for failure;
- Time of fitting PC treatment (PC1/PC2);
- Jaw (upper/lower jaw);
- Type of tooth (anterior/premolar/molar);
- Bone attachment (physiological: >75%/pathological: <75%);
- Luting material (conventional cement/adhesive cement);
- PC material (high-gold-content alloy (hg)/non-precious alloy (np)/fibre-reinforced composite (fr));
- Therapist (dentist/student).

The patient cohort (n = 246) included 133 (54.1%) men and 113 (45.9%) women, with an average age of the patient at the time of fitting the PC treatment of 67.48 years (±10.45 years). The majority of treatments for PC were conducted by students under the strict supervision of experienced dentists (n = 314, 78.7%). Fewer treatments were conducted by the dentists themselves (n = 85, 21.3%). Both groups of therapists followed the same standardised workflow during post-space preparation, which is described by other authors as well [3,4]. A total of 267 (66.9%) PC were fitted after a fracture of an abutment tooth in order to reattach the existing TC (PC2), while 132 (33.1%) teeth were already treated with PC before the preparation for TCD treatment (PC1).

PC treatment was only performed if the tooth was free of symptoms indicating an inflammation or a root fracture [3]. Moreover, a sufficient circumferential ferrule of at least 2 mm of the TC was mandatory [11]. The decision whether a prefabricated fibre-reinforced post (PFRP) or a cast PC (CPC) was fitted was determined by the amount of remaining dentine and existing cavity walls [24,25]. A PFRP had been used if there were at least three walls left and CPC if the coronal destruction was more severe. Post-space preparation and impression for a CPC was performed according to a standardised and clinically established procedure [3,4]. In cases of PC2, the impression was taken using the TCD and TC as an impression tray in order to fix the position of the TC in relation to the abutment tooth. Regarding the different alloys, a non-precious PC had only been fitted if the TC was made of a non-precious alloy in order to prevent corrosion. In all other cases, a high-gold-content alloy was used. Before the fitting of a PC, the therapist evaluated the friction in the root canal and decided if a conventional cement (high friction of CPC) (N = 286/71.7%) or an adhesive cement (low friction of CPC/every PFRP according to the manufacturer’s advice) (N = 113/28.3%) had to be used. In cases of PC2, the TC was fitted at the same time as the CPC and the same sort of luting material was used for both restorations. If the PFRP was fitted as a PC2, the TCD and TC were used as a modelling aid for the composite core build-up by isolating the lumen of the TC with a thin layer of Vaseline. After removing the Vaseline, the TC was fitted using the same adhesive cement as for the PFRP fitting. In every case of PC2, the TCD was used as a positioning aid during the fitting process of the TC. In total, 190 (162 anterior teeth, 27 premolars and one molar) of the included PC treatments were in the upper jaw, whereas 209 (122 anterior teeth, 82 premolars and five molars) teeth were treated in the lower jaw.

Kaplan–Meier and Cox regression analyses were used to investigate the survival probability as well as possibly influencing co-parameters. Univariate influences were investigated by Kaplan–Meier and multivariate influences by Cox regression analysis, in which a reference variable is predefined in order to consider multiple influences on one co-parameter [26]. Significant differences between the subgroups of possibly influencing co-parameters were assessed by means of the log-rank test [27] with a significance level of *p* < 0.05. The evaluation was conducted by the forward stepwise logistic regression method based on the likelihood ratio, meaning that only parameters that had significant influences (x^2^: *p* < 0.05) on the failure probability were included in this analysis. Multiple PC treatments in the same patient were statistically considered using “shared frailty” [28].

## 3. Results

The overall average survival time of all cases included in this study was 6.96 years with a standard deviation (SD) of 0.36 and a 95% confidence interval (CI) of 6.25–7.67. The most common cause of failure was loss of retention (N = 121/30.3%), followed by root fracture (N = 29/7.3%), periapical inflammation (N = 25/6.3%), secondary caries (N = 20/5.0%), periodontal bone loss (N = 20/5.0%) and post-fracture (N = 6/1.5%). Root fracture was only recorded with CPCs, whereas post-fracture occurred solely with PFRPs. Furthermore, decementation of PC treatment was most frequently reported with PFRPs. The average survival time of CPCs was 6.78 years (SD = 0.38; CI = 6.04–7.52), whereas the average survival time of PFRPs was 7.57 years (SD = 0.73; CI = 6.14–9.00).

**Figure 1:**
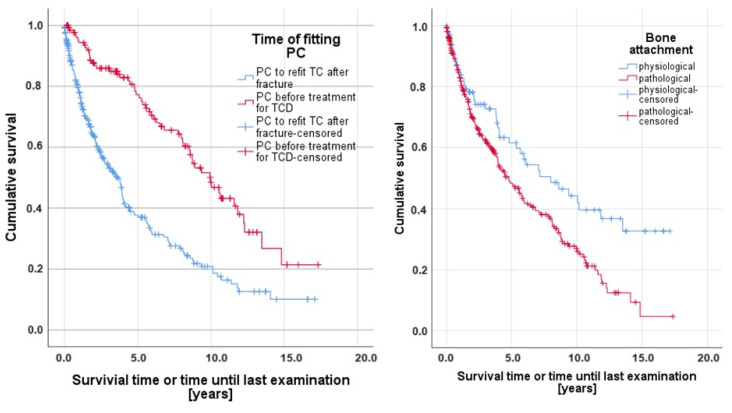
Kaplan–Meier survival curves of the significantly influencing parameters: time of fitting the PC treatment (**left**) and bone attachment (**right**).

The overall mean observation period was 4.13 years (SD = 4.04), with a maximum of 17.33 years. When distributing the cases according to the time of fitting the PC, the mean survival time for PC1 was 9.85 years (SD = 0.61; CI = 8.65–11.05), whereas PC2 survived for an average of 5.35 years (SD = 0.41; CI = 4.56–6.15). The log-rank test recorded highly significant differences (*p* < 0.001) between these two investigation groups (Table 1). Furthermore, the statistical analysis showed highly significant influences (*p* < 0.001) on the survival regarding the parameters “age of the patient at the time of fitting PC” as well as the “bone attachment” (Table 1 and Table 2). In detail, the risk for failure increased by 4.2% each year that the patient was older at the time of fitting the PC treatment, and the survival probability was inferior with pathological bone attachment. Figure 1 illustrates the Kaplan–Meier curves of the influencing parameters “Time of fitting PC” (left) and “Bone attachment” (right).

All other investigated co-parameters showed no significant influence (*p* > 0.05). Table 1 illustrates the results of the log-rank tests between the subgroups of the co-parameters.

Table 2 illustrates the results of the multivariate Cox regression analysis. The predefined reference variables of the co-parameters are written in brackets.

## 4. Discussion

The data had been automatically acquired by using a standardised protocol and a software (MZD), avoiding human mistakes and improving data correctness. Therefore, one can assume that the data are representative as well as comparable and they can be used to take a stand on the treatment option of fitting PC to reattach the TC after abutment tooth fracture (PC2) by comparison to the survival of PC that have been fitted before treatment for TCDs (PC1). Furthermore, a survival analysis including a large sample size with comparably long observation periods, up to 17 years as in the present study, would have been hardly possible in a prospective study. One reason for this is that it would have been ethically worrying to forgo PC treatment before fitting the TCD, waiting for abutment tooth fracture in order to fit the PC reattaching the TC. That is why we chose a retrospective study design, which is comparable to similar survival analyses in the scientific dental literature as well [3,5,29]. The present study resulted in highly significant lower survival rates when PC were fitted to reattach the TC after tooth fracture (PC2) in comparison to PC that have been fitted before treatment for TCDs (PC1). One reason for this might be possible microcracks in the root dentine in consequence of the previous abutment tooth fracture. Even if every tooth was visually checked for cracks, the dentist cannot exclude intraorally undetectable microfractures, which could enlarge during wear of the TCD after PC fitting. This enlargement of cracks could even be increased with a rigid CPC, because its mechanical properties might not match dentine, or when PC with a bad accuracy of fit have been inserted. In both cases, the extra axial forces on the abutment tooth with TCDs result in an uneven transmission of force into the dentine, leading to an increased risk of decementation and root fracture [15,16,17,18]. This is in line with the results of the present study because decementation and root fracture were the most common causes of failure and root fracture was only reported with CPCs. On the other hand, post-fracture was only observed with PFRPs. One reason for this might be the reduced mechanical stability of PFRPs compared to CPCs, which in connection with the extra axial forces may lead to higher post-fracture rates [16,18]. Recent studies using CAD/CAM technology described customised PC fabricated out of materials with matching mechanical properties to dentine [30,31,32]. These PC can reduce the risk for root fracture under extra axial forces [31]; thus, they might be a promising treatment option for TCDs because these PC treatments can combine the advantages of CPCs and PFRPs [31]. The influence of CAD/CAM PC treatments for the abutment teeth of TCDs should be further investigated in future studies. Rottner et al. described a new PC technique including a prefabricated PC with a ball attachment in order to reattach TCDs on the fractured abutment tooth rejecting the existing TC. They pointed out that the costs and the treatment time using their technique are less than for CPCs and comparable to established PFRPs [9]. Nevertheless, the observation period in this study was only three years and therefore not comparable to the long-term results of the present study. To the best knowledge of the authors, this is the only other survival analysis investigating PC survival after abutment tooth fracture of TCDs. Another disadvantage related to the technique described by Rottner et al. is that the ball attachment is limited to a strictly rigid connection to the TCD, transmitting occlusal forces only onto the tooth. However, TCDs with TCs can be modified in order to transmit occlusal forces onto both the gingiva and the abutment teeth, which can be advantageous for tooth preservation, especially in patients with few remaining teeth [7]. In this context, a possible limitation of the present study is that the number of abutment teeth for TCDs was not considered, since the chewing forces can be better distributed with an increasing number of abutment teeth [6,8]. This influence should be further investigated in future studies but was hard to consider in this retrospective setup because the number of abutment teeth was not constant in every patient during the whole observation period. In some cases, abutment teeth that were not treated with PC and therefore not included in this study had been extracted due to caries or periodontal bone loss; thus, the distribution of chewing forces on the remaining abutments had been changed. This influence is not considerable with the statistical evaluation methods used in the present study because the parameters in every case have to be constant. In order to decrease this influence on the data, only TCDs with at least two abutment teeth were included for data acquisition. In line with this limitation, the opposing dentition was not considerable with the used statistical methods because, due to further prosthetic treatments, the opposing dentition had changed in some cases during the observation period. A fixed dental prosthesis in the opposing jaw in comparison to removeable dentures can increase the chewing forces and can therefore have an influence on the survival of abutment teeth of TCDs treated with PC. This limitation has to be kept in mind when interpreting the results. Furthermore, the present study recorded significantly lower survival rates with pathological bone attachment and older patients at the time of fitting PC. A correlation of these parameters can be assumed, since the periodontal bone loss increases with the age of the patient [33]. In line with the results of the present study, Martino et al. described that the survival of PC treatment is significantly more likely if the bone attachment is above 75% [29].

Regardless of the influencing parameters, every patient that is treated with TCDs should participate in a strict aftercare in order to ensure the best survival of both the abutment teeth and the denture [8]. This aftercare is of even greater importance for TCDs than for other prosthetic restorations because an undetected and therefore untreated incongruent denture saddle increases the extra axial forces, and thus increases the risk for overloading the abutment teeth [6,8]. The results of the present study show that particularly with rigid CPC this can lead to an increased risk for root fracture.

## 5. Conclusions

The results of the present study show that PC treatment to refit TCs after abutment tooth fracture (PC2) should be well discussed with the patient because of the poor survival probability. If an abutment tooth is root filled and of high prosthetic value for TCD treatment, the dentist should take into consideration to fit the PC before TCD treatment (PC1) in order to increase the survival probability. Moreover, the bone attachment and the age of the patient should be considered in this decision-making process. In any case, one can lead from the literature and the results of the present study that patients treated with TCDs should follow a strict aftercare in order to prevent abutment teeth from excessive extra axial forces because of incongruent denture saddles. Furthermore, PC treatment options in connection to TCDs should be further developed because new digital technologies have already shown promising results that might be transferable to TCDs.

## Figures and Tables

**Table 1:** Results of the log-rank tests between the subgroups of the co-parameters.

Co-Parameter	Log-rank Test between Subgroups	*p*-Value
Time of fitting PC	PC1/PC2	<0.001 *
Gender	female/male	0.355
Jaw	upper/lower	0.405
Type of tooth	anterior/premolar	0.168
	premolar/molar	0.342
	anterior/molar	0.586
Bone attachment	>75%/<75%	<0.001 *
Luting material	conventional/adhesive	0.502
PC material	hg/np	0.217
	hg/fr	0.345
	np/fr	0.467
Therapist	dentist/student	0.791

* significant difference.

**Table 2:** Results of the multivariate Cox regression analysis (reference variable).

Co-Parameter	Subgroups (Reference Variable)	*p*-Value
Time of fitting PC (PC1)	PC2	<0.001 *
Gender (female)	male	0.630
Age at the time of fitting PC	/	<0.001 *
Jaw (upper)	lower	0.754
Type of tooth (anterior)	premolar	0.510
	molar	0.540
Bone attachment (>75%)	<75%	<0.001 *
Luting material (conventional)	adhesive	0.309
		
PC material (hg)	np	0.352
	fr	0.980
Therapist (dentist)	student	0.489

* significant difference.

## Data Availability

The data presented in this study are available on request from the corresponding author due to privacy and ethical restrictions.
